# Promoting evidence-based practice through peer teaching: Practical tips to run a departmental teaching program in a healthcare setting

**DOI:** 10.15694/mep.2020.000223.1

**Published:** 2020-10-08

**Authors:** Mercy Murinye Magwenzi

**Affiliations:** 1 University Hospitals Coventry and Warwickshire NHS Trust

**Keywords:** Medical education, Postgraduate medical education, Clinical teaching, Peer teaching, Evidence-based practice

## Abstract

This article was migrated. The article was marked as recommended.

In the face of knowledge explosion with new scientific evidence being published at an accelerated pace, doctors are faced with the challenge of keeping abreast with the relevant evidence to bring it to their patient. Evidence-based practice is core to providing the best care for patients and unawareness of new evidence against a certain practice in medicine can see the perpetuation of practices that are not evidence-based.

Peer teaching through departmental teaching programs is a useful tool to conceptualise evidence by bringing the patient into the classroom to consider the relevant evidence surrounding their care. Running a successful departmental teaching program which matches the intrinsic self-concept of clinicians who have multiple demands on their time is faced with multiple barriers. This article provides tips drawn from experience and supported by evidence from the literature that can be used in various healthcare settings to coordinate a departmental teaching program.

## Introduction

The practice of evidence-based medicine is at the centre of the provision of good patient care. A lack of awareness of said evidence for or against certain clinical practices contributes to continued practice which is not supported by evidence. Medical science is moving forward at an extremely fast pace and it is difficult for clinicians to keep up with this explosion of knowledge. A way to translate the relevant published evidence into daily practice is therefore important.

Departmental teaching programs are a useful tool to bring the data to the patient seen in practice. The constraints of a busy service, a large team with practitioners at different levels of their training and with varied experience and needs are some of the barriers to regular departmental teaching. Peer teaching works on the premise of cognitive congruence where the teacher is felt to be perceptive of the learner's needs ( Lockspeiser *et al*., 2006).

**Figure 1.**
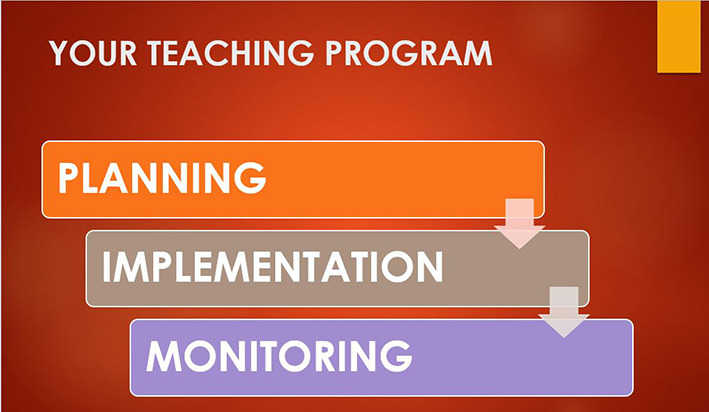
The 3 domains to run a teaching program

This paper provides practical tips on how to run a successful teaching program drawing from experience and relevant findings from the literature. The tips are set out in three domains: Planning, Implementation and Monitoring and apply to a variety of healthcare settings. Figure 1.

## Planning

### Perform a ‘pre-course’ survey

It is important to gather information about the department within which the teaching program is to be set up. The survey can be in the form of verbal canvassing or a formal survey questionnaire using a paper or online tool. The aim is to identify what is already happening within the department and determine whether a new teaching program is needed or there is one that can be built upon. The survey will also be useful to reveal the culture of the department by checking attitudes of practitioners to teaching. Determine whether there is an interest or focus is only on service provision. Ascertain if there is allocated time for teaching already and whether there are systems in place to facilitate on-call team members to attend teaching sessions. This knowledge will empower the next steps in the process.

### Assess the team and their needs

Consider who works in the department and what their learning needs are. A change in practice is more likely to occur when a needs assessment has been conducted. Think about these two scenarios, a general practice staffed by general practitioners and general practitioners-in-training and a general paediatrics department in a large teaching hospital which may have a team comprising of recently qualified doctors, paediatric trainees at different levels of training, consultant paediatricians, advanced nurse practitioners, physician associates, and trainees from other specialities who are doing their rotation in paediatrics for the experience. These teams are different, and the setup of the teaching should reflect this.

In designing formalised educational systems for professional improvement, a learning needs assessment should therefore be the starting point ( Grant, 2002). In the latter for example, it would be reasonable to have three people assigned to lead a teaching session at a time with a junior trainee doing the case presentation, a more senior trainee working on the literature review and a consultant to have oversight. This is essential for relevance to the wider team to meet individual training or continuous professional development needs as teaching sessions are then tailored to the audience.

### Occupy mind space

When a behaviour or activity is repeated within the same context, it becomes automatic to perform ( Lally *et al*., 2009). The goal is to make the teaching program a fixed fixture within the department. Even if the culture was not supportive of a teaching program the habitual nature of regularity will overcome this barrier. Decide on how often the teaching sessions should run. Weekly sessions are easier to remember, however, where it is not possible due to other demands then bimonthly would suffice. In the beginning, the attendance at the teaching sessions will be a cognitive process but as automaticity sets in through repetition, ( Lally, Wardle and Gardner, 2011) it follows that the sessions will be incorporated into the fabric of the department.

Using various forms of communication will help people to remember. Visual aids are particularly useful. Put up posters within the department announcing advance topics, venue and time of teaching sessions. Use social media within the limits of what is available; create and post colourful posters using web-based tools and word clouds that highlight the most important aspects. Knowing when to post on the department's social media groups for maximum exposure will maximise the value of the communication tools. Direct communication adds a personal touch; talk about it, send messages to individuals in your department and send weekly reminders via email which can be automated for simplicity.

### Set the scene

The venue of the teaching sessions may well be as important as the content of the sessions. It is important to consider who will be attending, how many people are expected to determine the size of the space required, facilities within the venue and whether they are suitable for the sessions. On-the-ward teaching is valuable for clinical skills teaching however there is a greater risk of interruptions when teaching is conducted on the wards. Classroom style of teaching in a lecture room or seminar room away from the ward need not be passive - the teaching program functions to bring the clinical cases to the classroom through case presentations. The venue should have the right equipment such as flipcharts and pens, whiteboard, computer, a projector, a good internet connection, depending on the healthcare setting.

Book the room in advance for the specific time for the teaching sessions in blocks of up to six months. Engagement will suffer if teaching sessions are missed because there is no suitable venue on the day. A constant location known by everyone will also help with occupying mind space by providing context for habit formation ( Lally *et al.*, 2009).

### Get organised

A well-organised teaching program has a better chance of success. Most clinicians work on a rotational system to cover out of hours' service and this needs to be accounted for when planning the teaching. Close collaboration with the work rota coordinators will help to avoid clashes with annual leave and off days. Establish a communication pattern for schedules of who is presenting, when they are presenting and what they are presenting on to enable sufficient time for preparation by the presenters and circulation of any pre-reading material.

There is value in presenters deciding the topic to be presented. The overall goal of the teaching program can be shared as a guide such as determining that all presentations work towards promoting evidence-based practice. Providing choice has been found to enhance intrinsic motivation, effort, task performance and perceived competence ( Patall, Cooper and Robinson, 2008). Without separable rewards, this enhanced tendency to be curious will lead to presenters aspiring to improve their skills and knowledge ( Di Domenico and Ryan, 2017) which they will transfer to their peers. The topics once decided are then shared with the rest of the department.

## Implementation

## Foster a sense of community

When the team feel that they belong to the relational community of a professional learning environment and they matter to one another with a shared goal of meeting each other's learning needs the team will be more inclined to participate in the teaching/learning process ( McMillan and Chavis, 1986).

Introducing ideas such as ‘Cake Tuesday’, ‘Tester Wednesday’ where one of the presenters brings a cake to share with the rest of the team can make the teaching sessions fun whilst building a sense of community. Free-flowing refreshments in the form of tea and coffee also contribute to enhancing the experience at the sessions. Decide who buys the refreshments or create a pool of funds that everyone contributes to that can be used to purchase the refreshments. One-minute quizzes with token prizes on non-medical topics make an excellent ice- breaker.

## Aim for engaging sessions

Keeping learners engaged should be a goal during teaching sessions. As a teaching session progresses there can be inference when it becomes difficult to process new information whilst trying to assimilate what has been taught at the beginning of a session ( Cowan, 2008). Active learning strategies can therefore be used to overcome these issues. Buzz groups or breakout sessions provide a necessary interlude in a teaching session. Most teaching sessions allow for 45mins to 1hour and interactive learning will compensate for the length of the sessions without falling into the fallacy of assumed knowledge.

The need to know is central to adult learning and case-based presentations contextualize the teaching whilst also bringing the patients into the classroom creating the relevance necessary for improving evidence-based practice. Using cases that are on the wards as the basis for teaching ensures that teaching sessions will align with participants' intrinsic self-concept. With numerous demands on time for clinicians, the teaching sessions need to be perceived as compatible with knowledge gaps that need filling ( Cooper and Richards, 2017).

Build on experience in the room by asking presenters to draw out what participants already know about the topic and having the seniors share from their experiences by presenting opportunities for discussion. The choice of teaching method will depend on the presenters and the most engaging sessions are those that incorporate more than one.

## Facilitate dissemination of information

It is through effective dissemination strategies that acquired knowledge is shared. Once a teaching session is complete, the key messages for change of practice need to be shared with the wider team members as not everyone will be able to attend all teaching sessions. Dissemination is essential for uptake and some of the ways that can be used to disseminate information include posters, mass emails and messages on social media sites. This process forms part of the process for change ( Brewer, 2016); knowledge is established, persuasion occurs in the presentation, the decision for adoption made and disseminated for implementation. The presentations can also be kept on a local database accessible to everyone for future reference; an alternative to this would be the use of handouts with summary points.

## Keep the program fresh

Inviting guest speakers from within the multidisciplinary team will break the monotony of peer-peer teaching however useful it may be. Guest speakers will bring a fresh perspective and being specialists in their area they can also improve the overall learning experience. The element of choice and autonomy is once again vital; the allocated presenters for a teaching session can select and invite the speaker if the vision for the teaching program is met. It is important to communicate what the overall program is trying to accomplish and sometimes go through what the speaker intends to talk about to avoid repetition ( Zou *et al*., 2019) and to ensure there is no opportunity to further own agenda.

A multidisciplinary presence also offers an opportunity for networking and developing an understanding of how other teams work. A database of guest speakers will reduce the work for finding appropriate speakers ( Zou *et al*., 2019).

## Be ready to adapt

The very nature of life bellies the need for an attitude that embraces change, challenges, and uncertainty ( Margolis, 2018). This holds in organising a teaching program. A teaching venue may be inadvertently double-booked at the same time as a session and this requires on the go thinking and finding solutions. Remaining calm in the face of uncertainty is crucial. Clinical pressures or changes in the way the department works may warrant a change to an online venue when participants cannot be in the same place or the presenter is off-site. The technology to support this will need to be sought and use of web-based click tools for participation during the session will work to maintain interactive sessions.

## Monitoring

### Seek to improve

Work towards improving by obtaining feedback on the whole teaching program at regular intervals. Presenters should be encouraged to obtain feedback after each teaching session. The feedback will be a valuable instrument to gather information, consolidate awareness of the strengths of the program and identify areas of improvement ( Hardavella *et al*., 2017). Informal feedback can be sought through normal daily chat and actively listening to comments about the program. Obtain formal feedback through questionnaires asking direct questions about the program. Feedback is a form of communication and the loop is complete when the team is made aware of what improvements are being done to the program when the feedback has been gathered. This process will build on the sense of community and a realisation that their opinions are valued.

### Measure the impact of the program

After the clinical practice has been brought to the classroom and evidence for implementation has been discussed it is important to determine how well the learning is translating into daily practice. Various mechanisms can be employed in this process, for instance, informal assessment from other colleagues in the multidisciplinary team such as the ward pharmacist who may comment on a change in the first line antibiotic prescriptions occurring after a teaching session on the new guidance on antibiotic choice for urinary tract infections. A formal audit of practice can be performed by the wider team as part of quality improvement whilst also feeding into an assessment of how well the learning is having an impact on practice. Evaluation tools should align with the expected outcomes from each training session.

## Conclusion

Departmental teaching programs are a vital tool to drive the evidence-based practice of medicine through peer teaching. A successful program will meet the needs of the various clinicians at different training levels and its impact on clinical practice can be evaluated. The pointers provided here offer a guide to any clinician who has the task of running a teaching program in their department. The tips are mostly applicable in any healthcare setting and some adjustments can be made to suit as they are not by all means exhaustive.

## Take Home Messages

• Peer teaching through departmental teaching programs is a valuable tool in improving clinical practice
• Know the team that you will be coordinating, aim to understand their needs
• Encourage ownership of the program by building a sense of community
• Maintain interest through engaging presentations and multidisciplinary team involvement
• Use feedback to improve the program
• Evaluate the impact of the program on clinical practice

## Notes On Contributors

**Dr Mercy Murinye Magwenzi *,*** MBCHB, MRCPCH, is a Registrar in Paediatrics at the University Hospital of Coventry and Warwickshire NHS Trust, West Midlands, UK. She has interests in undergraduate and postgraduate medical education and training.
