# Supplementary material for: Promoting evidence-based practice through peer teaching: Practical tips to run a departmental teaching program in a healthcare setting
Source: MedEdPublish (2016). 2020 Oct 8;9:223. [Version 1] doi: 10.15694/mep.2020.000223.1 (PMC10699359; doi:10.15694/mep.2020.000223.1)
Supplement: Peer review history

# Reviewer report: Reviewer response for version 1

**DOI:** 10.21956/mep.20182.r31211

**Published:** 2020-10-13

**Authors:** Leila Niemi-Murola

**Affiliations:** 1 University of Helsinki

This review has been migrated. The reviewer awarded 4 stars out of 5 Thank you for the opportunity to review this interesting article, which gives practical tips for those interested in organizing departmental teaching sessions for overloaded clinicians. It is true that medicine is moving fast and it is hard to keep up with all relevant literature. However, there will always be everyday practices not being important enough to merit scientific research. However, there is always a need for scholarly approach and there might be new relevant findings in the literature. Thus, the three levels suggested by the author, namely Planning, Implementation of Monitoring are of interest to all clinicians.The Monitoring section was interesting, because the impact of clinical meetings is often taken as self-evident. Regular meetings are often important for the community but, with a little effort, they could be valuable learning opportunities as well. As the author points out, the tools used to measure the effect should be aligned with the intended learning outcomes of the session (knowledge, skills or attitudes, competencies or levels of Bloom’s taxonomy).This paper offers a valuable starting point for those who plan to start a tradition of regular learing session for clinicians and a check-list for those who already have such a program.

Reviewer Expertise:

NA

No decision status is available

# Reviewer report: Reviewer response for version 1

**DOI:** 10.21956/mep.20182.r31209

**Published:** 2020-10-12

**Authors:** Mohamed Al-Eraky

**Affiliations:** 1 University of Dammam

This review has been migrated. The reviewer awarded 3 stars out of 5 This is an interesting paper on how to plan and implement an independent teacher training program among peers of the same department. The innovative point in the approach that peers know what works or not in their context. That’s why they can offer relevant, reasonable and feasible comments and tips for their practice. Also, it’s nice to make the teaching program a fixed fixture within the department, as the author mentioned. The only challenge is to plan the program, not only based on interests of staff, but to acknowledge three other inputs to the plan, as indicated in the Compass Model (Figure 1) in here: https://drive.google.com/file/d/0ByugEmLvxFlUaGQydTBBX0h2czQ/view?usp=sharing There is a tricky point in needs assessment. We should fulfill the actual needs,not wishes. That’s why surveys are not enough, we need valid assessment of the performance of potential participants to be considered in planning and implementation.Teaching, learning and assessment are integral roles in the job description of faculty members, along with service provision. The program should also follow the institutional mission and educational strategies that are adopted, e.g. PBL, integration and the plan should be aligned with these directions. Finally, with respect to monitoring, it’s nice to solicit feedback from participants, peers and members of multidisciplinary team, yet we should not forget the direct beneficiaries of the program, namely: learners, students and residents. I would propose to develop a list of key performance indicators (KPIs) to evaluate the program and eventually perhaps designing a dashboard for program managers to monitor its progress over time. Good luck.

Reviewer Expertise:

NA

No decision status is available

# Reviewer report: Reviewer response for version 1

**DOI:** 10.21956/mep.20182.r31210

**Published:** 2020-10-09

**Authors:** Subha Ramani

**Affiliations:** 1 Harvard Medical School, Brigham and Women's Hospital

This review has been migrated. The reviewer awarded 4 stars out of 5 I enjoyed reading this paper as one of those busy clinicians within an academic department. Abstract- I agree that knowledge explosion just does not allow individual clinicians to keep abreast of rapidly changing clinical guidelines and protocols. How can one practice evidence based care if one does not know the latest evidence?It is very wise to think of busy clinicians and think of the educational format that will allow them to gain knowledge applicable at the point of care.Introduction-The first paragraph emphasizes the rapid pace of medical science which can be a barrier to evidence informed healthcare, this is true.Departmental teaching programs (CPD/CME) are essential because in these situations educational time is more or less protected for clinicians. I am not sure peer teaching is the only modality though. Self-study, point of care access to guidelines, expert presentations can also be valuable.Planning-The authors talk about the importance of needs assessment which includes examining the departmental culture, attitudes, time allocation etc as well as the needs of the staff themselves. Educational strategies and content are best when they are appropriate for the audience. Other tips such as embedding the program within the departmental routine, advertising the sessions and attention to the physical environment are all important tips.Implementation-Many steps under this heading showcase the value of community, engaging and interactive presentations and readiness to adapt to change. Though they seem intuitive, program developers often miss one or more of these steps.Monitoring-A continuous improvement mindset is critical for anyone designing educational programs. Feedback must be obtained from participants and leaders of such programs must be open to change. After all, if the program does not add value to the clinicians’ practice or motivate change, it cannot be called effective.Overall, this paper is well written, well referenced, the authors provide helpful tips for all educators designing educational programs for busy clinicians.I am not completely convinced that peer teaching is the main message here. Peer teaching can be one of the strategies but one does need experts in the field and some degree of self-directed learning.

Reviewer Expertise:

NA

No decision status is available
